# Seasonality in the incidence of anti‐GQ1b antibody syndrome—A territory‐wide study

**DOI:** 10.1002/brb3.2337

**Published:** 2021-09-06

**Authors:** Richard Shek‐kwan Chang, Eric H. Y. Lau, Elaine Yuen Ling Au, William C. Y. Leung, Yu Hin Ian Leung

**Affiliations:** ^1^ Division of Neurology, Department of Medicine, Queen Mary Hospital University of Hong Kong Hong Kong SAR China; ^2^ School of Public Health, Li Ka Shing Faculty of Medicine University of Hong Kong Hong Kong SAR China; ^3^ Division of Clinical Immunology, Department of Pathology, Queen Mary Hospital University of Hong Kong Hong Kong SAR China

**Keywords:** anti‐GQ1b antibody syndrome, epidemiology, Guillain–Barré syndrome, neuropathy

## Abstract

**Aims:**

To investigate any seasonality in the incidence of anti‐GQ1b antibody syndrome (AGS).

**Methods:**

We conducted a retrospective observational study in all hospitalized patients in local public hospitals from January 2013 to December 2018. AGS was defined by hospitalized patients with positive serum anti‐GQ1b IgG, presumably encompassing Miller‐Fisher syndrome, Bickerstaff brainstem encephalitis and Guillain–Barré syndrome (GBS) variants. GBS cases were retrieved from the computerized database by diagnostic label. *Campylobacter jejuni* infection (CJI) injection was identified by positive stool culture. Monthly incidence rates of AGS, GBS and CJI were calculated. Poisson and negative binomial regression models with long‐term time trend were fitted to characterize the seasonal pattern.

**Results:**

A total of 237, 572 and 2434 cases of AGS, GBS and CJI were identified, respectively, in a population of 7.3 million. The annual incidence rate of AGS was 0.54 per 100,000 person‐years. AGS was demonstrated to have an annual peak in the spring season, from March to April, which was congruent with that of GBS and slightly lagged the annual peak of CJI from February to March (likelihood ratio tests all *p* < .001 for the seasonal terms).

**Conclusion:**

The incidence of AGS peaks in springtime, which is congruent with that of GBS and lags around one month after that of CJI. We demonstrated that AGS has a clear seasonality in occurrence.

## 1 INTRODUCTION

Anti‐GQ1b syndrome (AGS) is a disease spectrum with positive serum anti‐GQ1b IgG antibody. It encompasses Miller‐Fisher syndrome (MFS), Bickerstaff brainstem encephalitis (BBE) and variants of Guillain–Barré syndrome (GBS) (Shahrizaila & Yuki, 2013). MFS is typified by a triad of external ophthalmoplegia, ataxia and areflexia (Fisher, 1956). BBE is characterized by external ophthalmoplegia, ataxia and altered consciousness (Bickerstaff & Cloake, 1951). Serum anti‐GQ1b IgG antibody has high sensitivity and specificity (Nishimoto et al., 2004; Yuki et al., 1993). The pathogenetic mechanism of anti‐GQ1b antibody production has been proposed to be molecular mimicry triggered by infections including *Campylobacter jejuni* (Kimoto et al., 2006).

Although seasonality has been previously described in GBS, epidemiology studies on AGS are relatively scarce. We designed a retrospective territory‐wide study to investigate any seasonal variation in the incidence of AGS.

## 2 METHODS

### 2.1 Data source

Data were retrieved from the central computerized database of Clinical Data Analysis and Reporting System (CDARS) of the Hong Kong Hospital Authority (HA). The HA is the sole operator of public hospitals of Hong Kong. It provides service through 42 public hospitals and covers about 90% of all secondary and tertiary care in a population of around 7.3 million (Authority, 2019). All hospitals use the International Classification of Diseases 9 (ICD9) code for encoding the diagnosis of each hospital admission. Other clinical information including patient demographics, hospitalization data and laboratory results are also recorded in CDARS (Cheung et al., 2004). A number of high‐quality, population‐based studies have been conducted based on this system (Chan et al., 2015; Chen et al., 2014; Chiu et al., 2002).

### 2.2 Case definition

AGS was defined as hospitalized patient with positive serum anti‐GQ1b IgG antibody, presumably included MFS, BBE and GBS variants. GBS diagnosis was commonly based on clinical, laboratory, including cerebrospinal fluid examination, and electrophysiological findings in our locality (Hui et al., 2005; Ma et al., 2010). Established nerve conduction study criteria were usually adopted (Hadden et al., 1998). As the AGS may have significant overlap with GBS, a subgroup analysis was performed on GBS patients without a positive anti‐GQ1b IgG antibody result (GBS/anti‐GQ1b‐). *C. jejuni* infection (CJI) was defined as any patient with at least one positive stool culture for the bacteria.

### 2.3 Study design

We conducted a retrospective territory‐wide study. Recruitment period was between January 1, 2013 and December 31, 2018. Only hospitalized patients were included. Eligible cases were patients from both pediatric and adult age groups. AGS cases were identified with “positive” or “strong positive” serum anti‐GQ1b antibody IgG test results; GBS cases were identified with a diagnostic label of “Guillain–Barré syndrome (ICD 357.0); and CJI cases were identified with a positive stool culture for *C. jejuni*. Repeated positive culture results within the same admission would be counted once only.

### 2.4 Laboratory investigation

Serum samples were collected from patients in all public hospitals in Hong Kong. The serum anti‐GQ1b antibody assay (GanglioCombi Light ELISA ‐ BUHLMANN, Switzerland) was then centrally performed at the Clinical Immunology laboratory of Queen Mary Hospital. The laboratory is accredited by the College of American Pathologists. The kit contains enzymes labels against IgG and IgM for the detection of anti‐GD1b, anti‐GQ1b and anti‐GM1. Stool cultures were performed by the microbiology laboratories of the corresponding hospitals.

### 2.5 Statistical analysis

Monthly incidences of AGS, GBS and CJI were calculated and analyzed. We extracted the mid‐year and year‐end Hong Kong population from 2012 to 2018 and performed spline interpolation to obtain the population size at each month. Poisson and negative binomial regression models with long‐term time trend and population size as offset term were fitted to characterize the seasonal patterns of AGS, GBS, CJI and GBS/anti‐GQ1b‐ incidences in Hong Kong. Specifically, we included harmonic terms in the model to test the potential seasonal pattern. The best model was selected using Akaike's Information Criterion (AIC). Statistical significance was established when *p *< .05. R version 4.0.2 (R Foundation for Statistical Computing, Vienna, Austria) was used for analysis.

## 3 RESULTS

A total of 237, 572, 481 and 2434 cases of AGS, GBS, GBS/anti‐GQ1b‐ and CJI were identified in the 6‐year period, respectively (Table 1). The incidence rates of AGS, GBS, GBS/anti‐GQ1b‐ and CJI were 0.54, 1.30, 1.10 and 5.56 per 100,000 person‐years, respectively, using an estimated average population of 7.3 million during the study period (Census and Statistics Department, 2019). Slight male predominance was demonstrated in all the four groups.

**TABLE 1:** Incidences and demographics of AGS, GBS and positive CJI

	AGS	GBS	GBS/anti‐GQ1b‐	CJI
Total cases	237	572	481	2434
Gender (M:F)	1.21:1	1.49:1	1.52:1	1.29:1
Mean age at diagnosis	53.6 (±19.67)	54.8 (±22.0)	54.7 (±22.69)	26.0 (±26.5)
Incidence rate (per 100,000 person‐years)	0.54	1.30	1.10	5.56

Abbreviations: AGS, anti‐GQ1b syndrome; CJI, *Campylobacter jejuni* infection; GBS, Guillain–Barré syndrome; GBS/anti‐GQ1b‐, Guillain–Barré syndrome without serum anti‐GQ1b IgG positivity.

**FIGURE 1:**
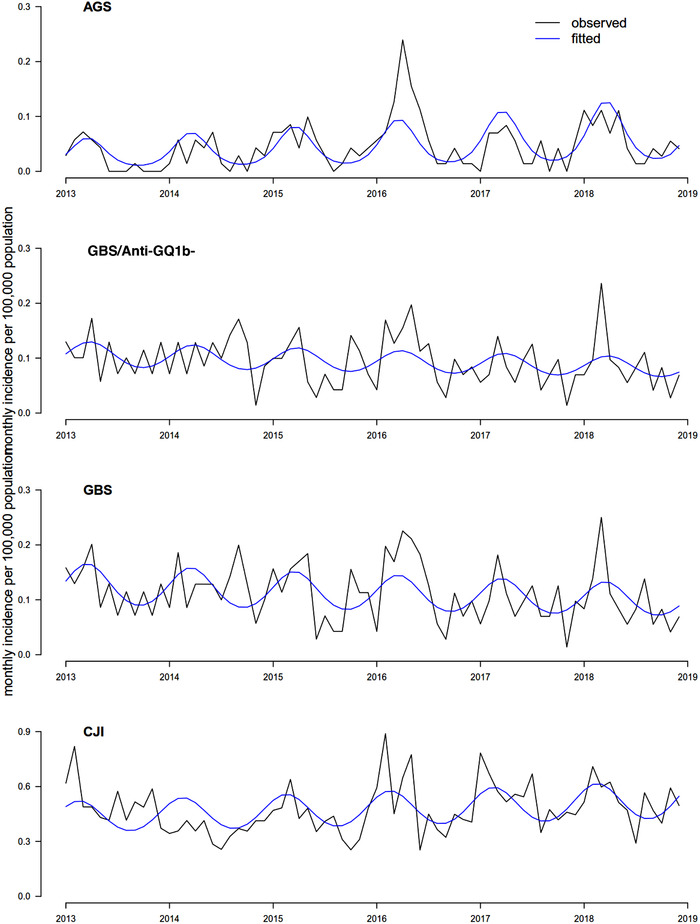
Observed and fitted monthly incidence of AGS, GBS and CJI. Abbreviations: AGS, anti‐GQ1b antibody syndrome; GBS, Guillain–Barré syndrome; GBS/anti‐GQ1b‐, Guillain–Barré syndrome without serum anti‐GQ1b IgG positivity; CJI, *Campylobacter jejuni* infection

Poisson regression and negative binomial models were fitted for AGS, GBS and CJI. Poisson regression model was selected for AGS and GBS, and negative binomial regression model was selected for CJI. The model fitted well for AGS and CJI but was less able to capture the more variable pattern of GBS (Figure 1). Long‐term incremental trend was found for AGS and CJI, with an increase of 1.2% (*p *< .001) and 0.3% (*p *= .012) per month, respectively. Clear seasonal pattern was identified for all three disease entities (likelihood ratio test, all *p *< .001 for the seasonal terms) (Figure 1). The peak months were identified to be from March to April for AGS and GBS, and from February to March for CJI, which are springtime in Hong Kong. A similar seasonal trend was also observed in GBS without positive anti‐GQ1b antibody. A seasonal pattern with peak incidence in March to April was also observed for GBS/anti‐GQ1b‐ (*p *< .005).

## 4 DISCUSSION

MFS and BBE have been considered as variants of GBS long before the discovery of anti‐GQ1b antibody (Bickerstaff, 1957; Fisher, 1956). The association was largely based on clinical presentations. GBS, MFS and BBE share common features of weakness, sensory deficit, areflexia and albuminocytological dissociation in cerebrospinal fluid. Subsequent discovery of anti‐GQ1b IgG antibody provided more insights into the pathomechanism of the disease spectrum (Chiba et al., 1993; Yuki et al., 1993). The term “Anti‐GQ1b antibody syndrome” was coined to describe demyelinating neuropathy in addition to various degrees of central nervous system involvement with positive anti‐GQ1b IgG, including MFS, BBE and certain GBS variants (Odaka et al., 2001). Molecular mimicry plays a key role. A wide range of infective agents bearing the GQ1b epitope, such as *C. jejuni*, are capable of triggering the production of anti‐GQ1b IgG antibody (Chiba et al., 1993; Houliston et al., 2007; Kimoto et al., 2006; Yuki, 2001).

Our study showed that AGS was relatively common in our locality. The results are consistent with previous studies conducted in Taiwan and Japan (Lyu et al., 1997; Mori et al., 2001; Yuan et al., 2000). GBS and its variants have been reported to have winter predominance in other countries which may have resulted from the winter surge of prodromal infections (Stowe et al., 2009; Tang et al., 2017; Webb et al., 2015). Vaccination, especially influenza vaccine, may be a potentially rare cause of AGS and GBS. The neurological complications are supposed to emerge several weeks after injection (Breman & Hayner, 1984; Shoamanesh et al., 2011). The population‐wide annual flu vaccination program is launched annually in October by the Hong Kong government. The temporality cannot explain the monthly peaks in our findings.

An increasing trend in the incidences of CJI was observed over the years. This may be multifactorial. Global warming may enhance the activity of food borne pathogens, such as *Campylobacter*, although the evidence is not definite (Lake & Barker, 2018; Smith & Fazil, 2019). Environmental contamination, change in food processing and storage methods, household and kitchen cross‐contamination have been implied (Allos, 2001; Weinberger et al., 2013). The incidence of CJI peaked annually in springtime in our population. This is different from the West where the foodborne infection is more prevalent in summer (Louis et al., 2005). This discrepancy has been reported but the reason remains elusive (Ho & Wong, 1985; McGechie & TB, 1982). CJI has an annual peak just preceding those of the GBS and AGS. This suggests that *C. jejuni* may be a major trigger of GBS and AGS locally. Temporal relationship in annual seasonal trends among AGS, GBS and CJI may provide indirect evidence to support the molecular mimicry theory in the pathogenesis of the disease spectrum that includes GBS, MFS and BBE.

This study has certain limitations. The diagnosis of GBS may be under‐reported as it is not a statutory notifiable disease in our locality. Laboratory tests for anti‐GQ1b IgG antibody, though homogenously performed by a single centralized accredited laboratory, could still involve false negativity or positivity. We were unable to analyze MFS, BBE and GBS variants separately due to the limitations of the ICD9 coding system. Exclusion of nonhospitalized patients may underestimate AGS, GBS and CJI incidents as some of them may only have mild symptoms. The stool cultures performed by different hospital laboratories may vary in test accuracy. We have not included the numbers of positive *C. jejuni* stool culture among our AGS and GBS cases. The reason is that it is not a common practice for local neurologists to check *C. jejuni* stool culture as part of GBS and AGS workup. Many neurologists may consider the culture is of low yield at the time of neurological presentation, and the results can neither rule in nor rule out the diagnosis of GBS or AGS.

## 5 CONCLUSION

The incidence of AGS peaks in springtime, which is congruent with that of GBS and lags around one month after that of CJI in our locality. The temporal relationships among AGS, GBS and CJI allow better understanding in the disease spectrum mediated by an autoimmune process involving anti‐GQ1b IgG antibody.

## AUTHOR CONTRIBUTORS

Chang Richard Shek‐kwan and Lau Eric HY contributed equally to this study. Chang Richard Shek‐kwan designed and conceptualized study; drafted the manuscript for intellectual content. Lau Eric HY performed data analysis, drafted and revised the manuscript. Au Elaine Yuen Ling contributed to data collection, drafting the manuscript. Leung William CY and Leung Yu Hin Ian contributed to data collection and analysis, drafting the manuscript. All authors reviewed and approved the final version of the manuscript. The corresponding author attests that all the listed authors meet authorship criteria and that no others meeting the criteria have been omitted.

## CONFLICT OF INTEREST

The authors declare no conflict of interest.

### PEER REVIEW

The peer review history for this article is available at https://publons.com/publon/10.1002/brb3.2337
